# Cardioprotection with nebivolol in patients undergoing anthracyclines: a randomized placebo-controlled trial

**DOI:** 10.1093/cvr/cvae266

**Published:** 2025-01-22

**Authors:** Giulio Stefanini, Carmelo Carlo-Stella, Francesco Cannata, Mauro Chiarito, Stefano Figliozzi, Laura Novelli, Costanza Lisi, Sara Bombace, Federica Catapano, Eleonora Indolfi, Cristina Panico, Francesco Corrado, Giovanna Masci, Rita Mazza, Francesca Ricci, Lorenzo Monti, Giuseppe Ferrante, Bernhard Reimers, Armando Santoro, Marco Francone, Bruno R da Costa, Peter Jüni, Gianluigi Condorelli

**Affiliations:** Department of Biomedical Sciences, Humanitas University, Via Rita Levi Montalcini 4, 20090 Pieve Emanuele-Milan, Italy; IRCCS Humanitas Research Hospital, Via Manzoni 56, 20089 Rozzano-Milan, Italy; Department of Biomedical Sciences, Humanitas University, Via Rita Levi Montalcini 4, 20090 Pieve Emanuele-Milan, Italy; IRCCS Humanitas Research Hospital, Via Manzoni 56, 20089 Rozzano-Milan, Italy; Department of Biomedical Sciences, Humanitas University, Via Rita Levi Montalcini 4, 20090 Pieve Emanuele-Milan, Italy; IRCCS Humanitas Research Hospital, Via Manzoni 56, 20089 Rozzano-Milan, Italy; Department of Biomedical Sciences, Humanitas University, Via Rita Levi Montalcini 4, 20090 Pieve Emanuele-Milan, Italy; IRCCS Humanitas Research Hospital, Via Manzoni 56, 20089 Rozzano-Milan, Italy; Department of Biomedical Sciences, Humanitas University, Via Rita Levi Montalcini 4, 20090 Pieve Emanuele-Milan, Italy; IRCCS Humanitas Research Hospital, Via Manzoni 56, 20089 Rozzano-Milan, Italy; Department of Biomedical Sciences, Humanitas University, Via Rita Levi Montalcini 4, 20090 Pieve Emanuele-Milan, Italy; IRCCS Humanitas Research Hospital, Via Manzoni 56, 20089 Rozzano-Milan, Italy; Department of Biomedical Sciences, Humanitas University, Via Rita Levi Montalcini 4, 20090 Pieve Emanuele-Milan, Italy; IRCCS Humanitas Research Hospital, Via Manzoni 56, 20089 Rozzano-Milan, Italy; Department of Biomedical Sciences, Humanitas University, Via Rita Levi Montalcini 4, 20090 Pieve Emanuele-Milan, Italy; IRCCS Humanitas Research Hospital, Via Manzoni 56, 20089 Rozzano-Milan, Italy; Department of Biomedical Sciences, Humanitas University, Via Rita Levi Montalcini 4, 20090 Pieve Emanuele-Milan, Italy; IRCCS Humanitas Research Hospital, Via Manzoni 56, 20089 Rozzano-Milan, Italy; Department of Biomedical Sciences, Humanitas University, Via Rita Levi Montalcini 4, 20090 Pieve Emanuele-Milan, Italy; IRCCS Humanitas Research Hospital, Via Manzoni 56, 20089 Rozzano-Milan, Italy; Department of Biomedical Sciences, Humanitas University, Via Rita Levi Montalcini 4, 20090 Pieve Emanuele-Milan, Italy; IRCCS Humanitas Research Hospital, Via Manzoni 56, 20089 Rozzano-Milan, Italy; Department of Biomedical Sciences, Humanitas University, Via Rita Levi Montalcini 4, 20090 Pieve Emanuele-Milan, Italy; IRCCS Humanitas Research Hospital, Via Manzoni 56, 20089 Rozzano-Milan, Italy; Department of Biomedical Sciences, Humanitas University, Via Rita Levi Montalcini 4, 20090 Pieve Emanuele-Milan, Italy; IRCCS Humanitas Research Hospital, Via Manzoni 56, 20089 Rozzano-Milan, Italy; Department of Biomedical Sciences, Humanitas University, Via Rita Levi Montalcini 4, 20090 Pieve Emanuele-Milan, Italy; IRCCS Humanitas Research Hospital, Via Manzoni 56, 20089 Rozzano-Milan, Italy; Department of Biomedical Sciences, Humanitas University, Via Rita Levi Montalcini 4, 20090 Pieve Emanuele-Milan, Italy; IRCCS Humanitas Research Hospital, Via Manzoni 56, 20089 Rozzano-Milan, Italy; Department of Biomedical Sciences, Humanitas University, Via Rita Levi Montalcini 4, 20090 Pieve Emanuele-Milan, Italy; IRCCS Humanitas Research Hospital, Via Manzoni 56, 20089 Rozzano-Milan, Italy; Department of Biomedical Sciences, Humanitas University, Via Rita Levi Montalcini 4, 20090 Pieve Emanuele-Milan, Italy; IRCCS Humanitas Research Hospital, Via Manzoni 56, 20089 Rozzano-Milan, Italy; Department of Biomedical Sciences, Humanitas University, Via Rita Levi Montalcini 4, 20090 Pieve Emanuele-Milan, Italy; IRCCS Humanitas Research Hospital, Via Manzoni 56, 20089 Rozzano-Milan, Italy; Department of Biomedical Sciences, Humanitas University, Via Rita Levi Montalcini 4, 20090 Pieve Emanuele-Milan, Italy; IRCCS Humanitas Research Hospital, Via Manzoni 56, 20089 Rozzano-Milan, Italy; Department of Biomedical Sciences, Humanitas University, Via Rita Levi Montalcini 4, 20090 Pieve Emanuele-Milan, Italy; IRCCS Humanitas Research Hospital, Via Manzoni 56, 20089 Rozzano-Milan, Italy; Clinical Trial Service Unit and Epidemiological Studies Unit (CTSU), Nuffield Department of Population Health, University of Oxford, Oxford, UK; Clinical Trial Service Unit and Epidemiological Studies Unit (CTSU), Nuffield Department of Population Health, University of Oxford, Oxford, UK; Department of Biomedical Sciences, Humanitas University, Via Rita Levi Montalcini 4, 20090 Pieve Emanuele-Milan, Italy; IRCCS Humanitas Research Hospital, Via Manzoni 56, 20089 Rozzano-Milan, Italy

Chemotherapy with anthracyclines has revolutionized the management and outcomes of both solid tumours and haematologic malignancies.^1^ Notwithstanding, the use of anthracyclines may be associated with the onset of cancer therapy-related cardiac dysfunction (CTRCD), a fearsome complication with a significant impact on patients’ morbidity and mortality.^2^ Several small randomized trials and pooled analyses have been exploring the effectiveness of a primary prevention strategy—mainly based on neurohormonal antagonists—with heterogeneous findings.^3–6^ However, these trials had significant limitations, including a wide variability of risk profiles of included patients, an unblinded study design, and a left ventricular ejection fraction (LVEF) assessment relying only on echocardiography. Based on this relatively weak evidence, the European Society of Cardiology (ESC) guidelines established a Class of Recommendation IIa for the use of either renin–angiotensin–aldosterone system (RAAS) inhibitors or beta-blockers in high- and very high-risk patients receiving anthracyclines and solicited new trials exploring the controversial topic of primary prevention of CTRCD.^2^ Nebivolol-induced nitric oxide release has shown favourable effects in terms of antioxidant activity, cardiac neo-angiogenesis, and mitochondrial and endothelial protection. We, therefore, aimed to assess whether nebivolol is able to prevent the cardiotoxic effects of anthracyclines.^7–9^

The *evaluation of CardioprOtection by the use of betablocker Nebivolol in paTients with bReast cancer Or diffuse Large B cell lymphoma undergoing chemotherapy with anthracyclines: a randomized controlled trial* (CONTROL) was an individually randomized, parallel, placebo-controlled, double-blinded, superiority trial, aiming to assess the cardioprotective role of nebivolol in patients with breast cancer (BC) or diffuse large B cell lymphoma (DLBCL), scheduled for first-line chemotherapy with anthracyclines. A detailed description of the study methods is available online (https://zenodo.org/records/13936826). Only patients without a history of cardiac diseases and with a normal cardiac function as assessed by echocardiography were eligible for inclusion. The study complies with the Declaration of Helsinki and was conducted at IRCCS Humanitas Research Hospital (Rozzano, Milan, Italy) after approval by the local Ethics Committee (n. 012018). All patients signed a written informed consent. The study flow is summarized in (*Figure 1A*). Patients were randomly allocated, centrally using a web-based system, to nebivolol or an equivalent placebo in a 1:1 fashion. The allocation sequence was computer-generated in randomly varying blocks and stratified by type of malignancy (BC or DLBCL). All included patients underwent cardiac magnetic resonance (CMR) at baseline and at 12-month follow-up, and were evaluated with cardiological assessment, electrocardiography, echocardiography, and cardiac biomarker sampling at baseline, and at 1, 6, and 12 months of follow-up. The primary endpoint was LVEF reduction assessed by CMR at 12 months of follow-up. LVEF reduction was defined as the difference between LVEF at baseline and LVEF at 12 months follow-up (LVEF reduction = 12 months LVEF−baseline LVEF). The trial is registered in the EudraCT registry (number: 2017-004618-24) and in the ClinicalTrials.gov registry (identifier: NCT05728632). The study protocol has been published.^7^

**Figure 1:**
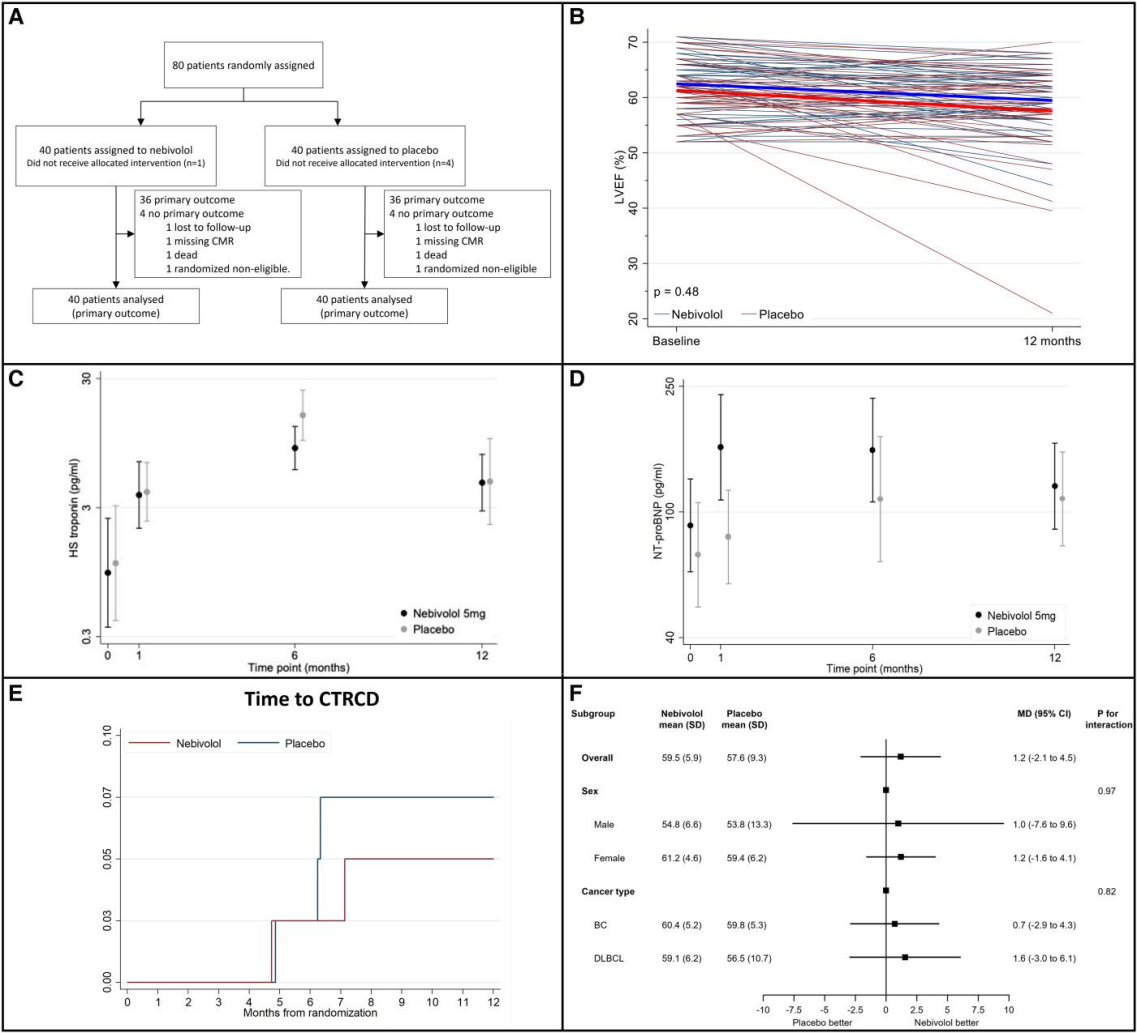
(*A*) Study flowchart. (*B*) The primary endpoint is LVEF reduction at 12 months, based on an ANCOVA model with LVEF at 12 months as dependent variable and treatment and LVEF at baseline as independent variables. (*C* and *D*) Troponin and NT-pro-BNP values over time were compared between groups using ANCOVA model. (*E*) Survival curve for CTRCD; compared between groups using a χ^2^ test. (*F*) Primary endpoint stratified by key subgroups; based on an ANCOVA model with LVEF at 12 months as dependent variable and treatment and LVEF at baseline as independent variables.

Between 3 January 2019 and 28 February 2022, a total of 80 patients with DLBCL (*N* = 55) or BC (*N* = 25) were randomized to nebivolol (*N* = 40) or placebo (*N* = 40). Baseline characteristics were comparable between groups. CMR assessment showed a similar LVEF at baseline in nebivolol (62.5 ± 5.6%) and placebo (61.3 ± 4.9%) groups. LVEF at 12 months was 59.5 ± 5.9% in patients allocated to nebivolol and 57.6 ± 9.3% in those allocated to placebo. The primary endpoint LVEF reduction at 12 months did not differ significantly between nebivolol and placebo (1.2%, 95% confidence interval −2.1 to 4.5%, *P* = 0.48; *Figure 1B*). Hs-Troponin values increased in both groups during chemotherapy and were similar between groups at 1, 6, and 12 months (*Figure 1C*) while N-terminal pro–B-type natriuretic peptide (NT-pro-BNP) and B-type natriuretic peptide were significantly different at 1 month but similar at 6 and 12 months (*Figure 1D*). All the other imaging parameters, assessed with echocardiography or CMR, were similar between groups at different time points. At 12 months, none of the patients allocated to nebivolol and two patients allocated to placebo died (*P* = 0.49). No cerebrovascular events or myocardial infarctions were recorded during the trial. Five patients developed a CTRCD, as defined by the ESC guidelines, without statistically significant differences between groups (*P* = 1.00; *Figure 1E*). The analyses of the primary outcome stratified by type of malignancy and sex did not find any statistically significant interaction (*Figure 1F*).

The key findings of the CONTROL trial can be summarized as follows: (i) the use of nebivolol did not confer an advantage in terms of LVEF reduction at 12-month follow-up as compared with the placebo group as well as in any of the secondary endpoints; (ii) the CTRCD risk is in line with existing literature, and the risk was not modified by the preventive use of nebivolol; (iii) the reduction in LVEF at 12 months is modest in patients undergoing chemotherapy with anthracyclines without a history of cardiac diseases and with a normal LVEF at baseline.

The neutral results of this study are in contrast with those of previous evidence suggesting a statistically significant benefit of neurohormonal antagonists in terms of prevention of anthracycline-induced LVEF reduction, albeit without an impact on clinical outcomes.^3,5,6,10^ The CONTROL trial was conducted in a population with an overall low baseline cardiovascular risk and receiving contemporary doses of anthracyclines, with a CMR-based assessment of LVEF and an active clinical follow-up. In this context, our trial provides a methodologically robust and consistent answer to the open question on the role of primary prevention in low-risk patients undergoing chemotherapy with anthracyclines and supports the current ESC guidelines.^2^ To resolve this controversy, it may be necessary to reconsider the concept of primary prevention of CTRCD with neurohormonal antagonists. Indeed, the initial mechanisms underlying the anthracycline-induced CTRCD—involving oxidative stress, mitochondrial dysfunction, and impaired calcium handling—may not be prevented by the use of a neurohormonal antagonist. Anthracycline-related neurohormonal activation occurs only as a direct consequence of myocardial injury and correlates with the severity of subsequent ventricular dysfunction and heart failure development. When the RAAS is activated, the myocardial injury has already occurred, and the process leading to CTRCD and heart failure has been unavoidably triggered. The use of RAAS inhibitors or beta-blockers in patients undergoing anthracycline chemotherapy should not represent a primary prevention strategy but rather a treatment for patients who develop left ventricular dysfunction. Neurohormonal inhibition targets a potential consequence of the cardiotoxicity rather than the cardiotoxic injury *per se*. Therefore, if we put in context the current evidence on CTRCD prevention in this new background, we can easily understand the disappointing results in low-risk patients of a strategy based on the treatment of left ventricular dysfunction even in absence of the dysfunction itself.

A number of limitations should be acknowledged. First, the sample size is relatively small. However, the strength of our findings relies on the rigorous methodology for CMR-based LVEF assessment, as well as the consistent primary and secondary outcomes with no signal suggesting any potential treatment effects. Secondly, the single-centre nature of the study might limit the generalizability of our findings. Thirdly, as a result of the inclusion criteria, most of the randomized patients were female, representing 70% of the population. However, while being a potential limitation for generalizability in male patients, this characteristic renders the present study one of the first cardiovascular randomized trials with a prevalent female representation.

In conclusion, in patients with BC or DLBCL undergoing first-line chemotherapy with anthracyclines, nebivolol does not provide clinically meaningful cardioprotective effects at 12-month follow-up.

## Data Availability

The data underlying this article cannot be shared publicly for the privacy of individuals that participated in the study. The data will be shared on reasonable request to the corresponding author.
